# Moxifloxacin Monotherapy in Left-Sided *Staphylococcus aureus* Endocarditis

**DOI:** 10.1155/2021/5586450

**Published:** 2021-04-14

**Authors:** Yucel Colkesen

**Affiliations:** Department of Cardiology, Erdem Hospital, Istanbul, Turkey

## Abstract

*Staphylococcus aureus* is the major cause of endocarditis, and its mortality has remained high despite therapeutic procedures over time. A case of left-sided native valve endocarditis caused by methicillin-sensitive *Staphylococcus aureus* which responded well to moxifloxacin monotherapy is described. An 83-year-old woman with a history of current hospitalization presented with fatigue and fever. Transthoracic echocardiography depicted vegetation, and blood cultures were positive for *Staphylococcus aureus*. After a 14-day intravenous administration of moxifloxacin, a good clinical response was achieved, and antibiotic regimen transitioned to oral moxifloxacin for an additional four-week therapy.

## 1. Introduction

*Staphylococcus aureus* (*S. Aureus*), being the most common cause of infective endocarditis, (IE) is an intractable microorganism [1, 2]. Infection may scatter rapidly resulting in multiple cardiac and noncardiac complications such as valve destruction and insufficiency, heart failure, septic emboli, pericarditis with effusion, and mycotic aneurysm of the coronary arteries [3]. Treatment is still a challenging problem and usually requires at least two parenteral antibiotics for left-sided IE with a prolonged duration up to six weeks depending on the severity [2]. Nevertheless, mortality and morbidity remains high. To facilitate the therapy, newer antibiotics and novel regimens are being tested in this regard [4, 5].

## 2. Case Report

A 83-year-old woman with a history of chronic atrial fibrillation presented to an outpatient clinic with fatigue and fever. She has been hospitalized a week before her admission due to decompensated cardiac failure and given intravenous diuretics. On physical examination, her body temperature was 38.7°C, pulse rate was 110 beats/minute, blood pressure was 140/80 mmHg, and respiratory rate was 18 breaths/minute with an oxygen saturation of 96% on ambient air. Overall, the patient appeared weak. Auscultatory exam revealed irregularly irregular heart beats and a 2/6 holosystolic murmur at the apex and a 1/6 diastolic murmur at the left lower sternal border. She had minimal left pulmonary crackles on inspiration. There were no digital clubbing, Osler's nodes, or Roth's spots. Jugular venous pressure was normal, and there was no peripheral edema. Because of her previous stay, a hospital-acquired infection was suspected along with COVID-19. Hence, empirical parenteral antibiotics moxifloxacin (MOX) (400 mg once daily) and cefoperazone-sulbactam combination (1.0 g + 1.0 g twice daily) were administrated immediately. The decision was awaited until the laboratory test results.

**Figure 1:**
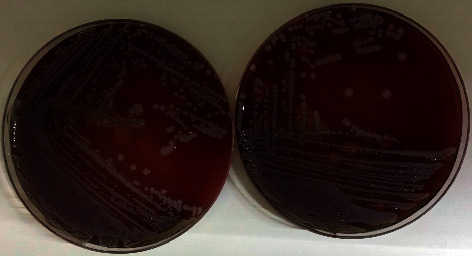
*Staphylococcus aureus* on blood agar.

Electrocardiogram was compatible with atrial fibrillation. No obvious infiltrates on chest X-ray were seen. Her laboratory data included hemoglobin of 10.7 g/dL, white blood cell count of 21,060 cells/mm^3^ with 94.6% of segment form, a platelet count of 307.000/uL, and C-reactive protein of 56.6 mg/dL. A real-time polymerase chain reaction assay performed on a nasopharyngeal swab specimen was negative for COVID-19. Prior to initiation of empirical regimen, blood samples were taken for culture from different venipuncture sites. On the second day of moxifloxacin therapy, the patient's fever returned to normal. Subsequently, test results for blood cultures came positive for methicillin-sensitive *S. aureus* that was sensitive to quinolones (Figure 1) (Table 1). Transthoracic echocardiography (TTE) was performed to confirm the diagnosis of IE. Notable echocardiographic findings included aortic valve echo density, mild aortic regurgitation, calcified posterior mitral leaflet, and moderate mitral insufficiency. TTE revealed mobile vegetation attached to atrial aspect of the posterior leaflet of mitral valve (Supplementary video). Ejection fraction was estimated at 63%, and there was moderate to severe mitral regurgitation. Cefoperazone-sulbactam was stopped on day 2.

**Figure 2:**
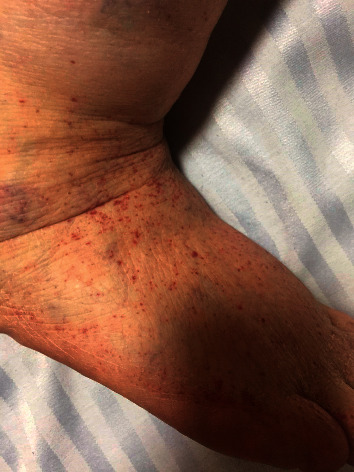
Janeway lesions: a sign of septic microembolism.

In addition to resolution of fever, improvement in general condition of the patient and a decrease in the number of leukocytes motivated the decision to continue solely with parenteral MOX. Meanwhile, Janeway lesions appeared on both legs (Figure 2). On day 3 of antibiotic therapy, the white blood cell count returned to normal and the patient had no fever. During a 2-week hospitalization, the patient had continuous parenteral MOX therapy (400 mg once daily). By hospital day 14, blood cultures and other infectious disease workup proved to be negative. The patient's hospital course was uneventful and fortunately uncomplicated. No residual vegetation was assessed by TTE at discharge. She was prescribed an additional four-week postdischarge course of oral MOX 400 mg once a day. At her three and six month follow-ups, the patient was asymptomatic, and TTE did not reveal any pathology pertaining infective endocarditis (IE).

## 3. Discussion

Initial antibiotic treatment in infectious diseases is generally empirical because the determination of a pathogen may take days from time to time [1]. Both ESC and ACC/AHA guidelines do not recommend quinolones as first-line therapy in IE [1, 3]. On the contrary, in recent studies a trend toward quinolone monotherapy has been observed in uncomplicated cases. Clinical outcomes were convincing in these reports [4, 5].

Moxifloxacin has a prolonged serum half-life, broad spectrum of antimicrobial activity, and good safety features such as excellent absorption, single daily dosing, and good tolerability. MOX belongs to the fourth generation of quinolone antibiotics. In selected patients, thanks to its high activity against Gram-positive bacteria, MOX may be preferred as first-line strategy [6]. In vivo experimental analysis of MOX activity against *S. Aureus* shows that, with standard oral doses, MOX is both inhibitory and bactericidal against strains of *S. Aureus* [7]. In animal models, it is equally effective as vancomycin in the treatment of experimental aortic valve IE due to methicillin-resistant *S. aureus* [8]. In vitro experiments showed that 95% of culture-negative vegetations have been achieved in oral MOX treatment [9], and all parenteral MOX groups had sterile vegetations at the end of treatment plan. The cure rate was 100%, and no relapses of IE occurred after 5 days of therapy [8]. Strains of methicillin-resistant *S. aureus* were not resistant to MOX. Unless a high ciprofloxacin resistance existed, MOX treatment did not fail when compared to ciprofloxacin and vancomycin [9].

Duration of therapy should be up to six weeks in *S. aureus* IE due to well-established high bacterial densities within vegetation. Depending on the bactericidal activity of the selected regimen, a shorter duration of antimicrobial therapy only in unique instances with uncomplicated IE may be suggested [1]. According to guidelines, a 2-week treatment and oral therapy is limited only for uncomplicated right-sided native valve methicillin-susceptible *S. aureus* IE. Although these regimens are not allowed to be utilized in left-sided IE [3], recent studies offer an alternative treatment route to conservative management [4, 5].

One cohort study [4] and a randomized clinical trial [5] demonstrated that switching to oral antibiotics was not associated with increased mortality and was noninferior to continued intravenous treatment in patients with left-sided IE. All cases were shifted to oral regimens after an adequate response to initial intravenous antibiotics. In these studies, MOX was either given after the initial parenteral treatment [4] or as a part of a combination therapy [5].

## 4. Conclusion

Treatment of *S. aureus* IE with a 4th generation quinolone antibiotic with simplicity is the key message in this report. This case is the 1st reported case of sequential intravenous and oral MOX monotherapy as first-line treatment of left-sided native valve IE caused by methicillin-sensitive *S. Aureus*.

## Figures and Tables

**Table 1:** Antibiogram from *Staphylococcus aureus* strains.

Antibiotics	
Gentamicin	S
Trimethoprim/sulfamethoxazole	S
Tetracycline	S
Teicoplanin	S
Vancomycin	S
Levofloxacin	S
Clindamycin	S
Erythromycin	S
Linezolid	S
Ciprofloxacin	S
Penicillin	R
Methicillin	S

Zone diameter distributions were measured on blood agar by using the European Committee on Antimicrobial Susceptibility Testing (EUCAST) definitions. Definitions of *S* and *R* were used as follows: S, susceptible, standard dosing regimen: a microorganism is categorised as “susceptible, standard dosing regimen,” when there is a high likelihood of therapeutic success using a standard dosing regimen of the agent. *R*: resistant: a microorganism is categorised as “resistant” when there is a high likelihood of therapeutic failure even when there is increased exposure.

## Data Availability

All type of data used to support the findings of this study are included within the article.

## Supplementary Materials

Supplementary Materials Video: mobile vegetation attached to the atrial aspect of the posterior leaflet of the mitral valve is seen on the parasternal long axis view of TTE.

## References

[1] Baddour L. M., Wilson W. R., Bayer A. S. (2015). Infective endocarditis in adults: diagnosis, antimicrobial therapy, and management of complications. *Circulation*.

[2] Anstead G. M., Cadena J., Javeri H. (2014). Treatment of infections due to resistant *Staphylococcus aureus*. *Methods in Molecular Biology*.

[3] Habib G., Lancellotti P., Antunes M. J. (2015). 2015 ESC Guidelines for the management of infective endocarditis. *European Heart Journal*.

[4] Mzabi A., Kernéis S., Richaud C., Podglajen I., Fernandez-Gerlinger M.-P., Mainardi J.-L. (2016). Switch to oral antibiotics in the treatment of infective endocarditis is not associated with increased risk of mortality in non-severely ill patients. *Clinical Microbiology and Infection*.

[5] Iversen K., Ihlemann N., Gill S. U. (2019). Partial oral versus intravenous antibiotic treatment of endocarditis. *New England Journal of Medicine*.

[6] Song J.-H., Ahmed A., Ariffin N. L. (2008). Treatment recommendations of hospital-acquired pneumonia in Asian countries: first consensus report by the Asian HAP Working Group. *American Journal of Infection Control*.

[7] Berrington A. W., Perry J. D., Gould F. K. (2001). Bactericidal activity of moxifloxacin against *Staphylococcus aureus*. *Clinical Microbiology and Infection*.

[8] Galani L., Pefanis A., Sakka V. (2009). Successful treatment with moxifloxacin of experimental aortic valve endocarditis due to methicillin-resistant *Staphylococcus aureus* (MRSA). *International Journal of Antimicrobial Agents*.

[9] Entenza J. M., Que Y. A., Vouillamoz J., Glauser M. P., Moreillon P. (2001). Efficacies of moxifloxacin, ciprofloxacin, and vancomycin against experimental endocarditis due to methicillin-ResistantStaphylococcus aureus expressing various degrees of ciprofloxacin resistance. *Antimicrobial Agents and Chemotherapy*.

